# Simulation Model of a Steelmaking–Continuous Casting Process Based on Dynamic-Operation Rules

**DOI:** 10.3390/ma17174352

**Published:** 2024-09-03

**Authors:** Xin Shao, Qing Liu, Hongzhi Chen, Jiangshan Zhang, Shan Gao, Shaoshuai Li

**Affiliations:** 1 State Key Laboratory of Advanced Metallurgy, University of Science and Technology Beijing, Beijing 100083, China; beike_shaoxin@126.com (X.S.); 15528938238@163.com (H.C.); zjsustb@163.com (J.Z.); 2 Engineering Research Center of MES Technology for Iron & Steel Production, Ministry of Education, Beijing 100083, China; 3 Laiwu Iron and Steel Group Yinshan Section Steel Co., Ltd., Jinan 271104, China; 13561700806@163.com (S.G.); muzilier@126.com (S.L.)

**Keywords:** steelmaking–continuous casting, dynamic-operation simulation, sequence-casting constraints, dynamic-operation rules, plant simulation

## Abstract

The steelmaking–continuous casting process (SCCP) is a complex manufacturing process which exhibits the distinct features of process manufacturing. The SCCP involves a variety of production elements, such as multiple process routes, a wide array of smelting and auxiliary devices, and a variety of raw and auxiliary materials. The production-simulation of SCCP holds a natural advantage in being able to accurately depict the intricate production behavior involved, and this serves as a crucial tool for optimizing the production operation of the SCCP. This paper thoroughly considers the various production elements involved in the SCCP, such as the fluctuation of the converter smelting cycle, fluctuation of heat weight, and ladle operation. Based on the Plant Simulation software platform, a dynamic simulation model of the SCCP is established and detailed descriptions are provided regarding the design of an SCCP using dynamic-operation rules. Additionally, a dynamic operational control program for the SCCP is developed using the SimTalk language, one which ensures the continuous operation of the caster in the SCCP, using the discrete simulation platform. The effectiveness of the proposed dynamic simulation model is verified by the total completion time of the production plan, the transfer time of the heat among the different processes, and the frequency of ladle turnover. The simulation’s results indicate that the dynamic simulation model has a satisfactory effect in simulating the actual production process. On this basis, the application effects of different schedules are compared and analyzed. Compared with a heuristic schedule, the optimized schedule based on the “furnace–machine coordinating” mode reduces the weighted value of total completion time by 8.7 min, reduces the weighted value of transfer waiting time by 45.5 min, and the number of rescheduling times is also reduced, demonstrating a better application effect and verifying the optimizing effect of the “furnace–machine coordinating” mode on the schedule.

## 1. Introduction

The steel manufacturing process is a typical “process” industry, with the steelmaking–continuous casting process (SCCP) being a complex quasi-continuous/batch production process involving a large number of high-temperature physicochemical reactions. The complexity of the SCCP primarily manifests in frequent variations in its dynamics during its operation [1,2,3]. This characteristic mainly originates from uncertainties relating to production raw materials, metallurgical mechanisms, production devices, operational controls, and other aspects. Therefore, it is crucial for steelmaking plants to handle these dynamic variations and ensure that the production process remains in a stable operation state in order to achieve high efficiency and low-consumption production.

A considerable amount of study has been conducted by previous researchers aiming to address these dynamic variations. The SCCP interface technology [4,5,6] emphasizes the connection-matching and coordination-buffering techniques found among the main metallurgical processes such as steelmaking, secondary metallurgy, and continuous casting (CC). Yang et al [7] further developed the quantitative evaluation model for the multi-process collaborative operation quality, including the evaluation of three dimensions: laminar flow operation level, processes matching level, and scheduling model availability. These aspects are critical for ensuring the smooth and stable operation of the SCCP. The operation-mode optimization, ladle cycling control, and crane running control have been identified as the three typical process interface technologies for the SCCP and have received significant attention [8]. Operation-mode optimization serves as an important foundation for SCCP mass flow control [9]. Based on the optimization of operational modes, Liu et al. [10], Yang et al. [11], and Yuan et al. [12] have, respectively, improved the classical genetic algorithm, heuristic algorithm, and fast elitist non-dominated sorting genetic algorithm, which has significantly enhanced the algorithms’ performance in solving the static scheduling problem of the SCCP. In tackling the SCCP dynamic-scheduling problem, Tang et al. [13] considered the constraints of the practical technological requirements and their dynamic nature to model the SCCP dynamic problem and designed a new mutation strategy and additional incremental mechanisms, achieving a superior solution compared to other algorithms. Long et al. [14] developed a dynamic model capable of generating a new schedule scheme for each strategy by analyzing the impacts of different reallocation strategies on the dynamic-scheduling process and designed a hybrid algorithm featuring a genetic algorithm combined with a general variable neighborhood search, which is a better solution to the problem of continuous caster failures. In addition, ladle and crane control are also important parts of the SCCP operation control. Hong et al. [15] addressed the devices’ conflicts through the “ladle temperature drop control” strategy and proposed an enhanced genetic algorithm to solve the empty-ladle scheduling problem in steelmaking plants, which reduced the unreasonable ladle temperature drop and improved the “red-ladle” utilization ratio. Liu et al. [16] extracted ladle matching rules by rule-based reasoning and developed an intelligent re-scheduling method for the ladle based on a BP neural network working-condition estimation, which improved the SCCP production efficiency. Li et al. [17] presented a solution for a multi-crane scheduling problem by designing the trajectory plan for the cranes’ motion control based on the job prediction. This solution effectively improved the efficiency of crane operations in steelmaking plants. Kuyama et al. [18] developed a crane guidance system with scheduling optimization technology by employing a two-stage optimization approach that combines scheduling optimization and logistics simulation. This system effectively reduced the workload of cranes and facilitated efficient logistics in the steelmaking plants.

The above studies have thoroughly investigated the modeling and solution of scheduling problems, as well as the optimization of ladle and crane operations. However, most of the studies in this area have only focused on individual production factors, an approach which still exhibits limitations in practical on-site applications. This is due to the fact that SCCP is a complex, integrated production system in which close coordination among these production elements is essential. Any misalignment in the coordination among these elements disrupts the production process. Simulation modeling of the production process is a proven method for solving such problems. Intelligent modeling of complex industrial systems is one of the key issues that urgently needs to be addressed to achieve high-end, green, and intelligent developments within the process industry [19]. Production-process simulation can comprehensively consider the production elements of the factory and establish a digitally simulated factory that can precisely emulate the real-world operation conditions by reconstructing the logical relationships within complex systems. This serves as a crucial method for studying dynamic production processes [20,21]. Ramírez-López et al. [22] developed a simulator for the continuous casting process, investigating the effects of different strand numbers and casting speeds on production efficiency in detail, but their research was confined to continuous casting, and did not account for the influences of other processes on production. In the SCCP, one ladle carrying molten steel is sent to various processes for processing; this is the smallest unit handled by each process, also called a heat. Deng et al. [23] integrated the operations of multiple processes and developed a logistics simulation model for SCCP by tackling the challenge of sequence-casting by gathering a specified number of heats prior to the start of casting on the caster. Yang et al. [24] employed the strategy of process-route reversal to develop a SCCP simulation model under the crane non-collision constraint. The model’s reliability was assessed using evaluation indicators such as total completion time, ladle turnover frequency, and the transfer time of the heat among the different processes. Fanti et al. [25] utilized Unified Modeling Language (UML) to provide a detailed description of the manufacturing system activities in steelmaking plants and employed discrete-event simulation to validate the dynamics and performance of the SCCP system. Wei et al. [26] established an SCCP production-simulation model based on the Flexsim simulation software platform, disregarding the constraints of sequence casting, and analyzed the actual production process using the continuity degree, device utilization rate, and process stability as evaluation indicators. It is evident that the aforementioned studies employed various idealizations to simplify the simulation modeling and satisfy the sequence-casting constraints, such as strategies like heat gathering before casting, process-route reversal, and discretization, etc. However, there are some imperfections in these idealizations, which resulted in certain limitations and discrepancies between the simulation results and actual production situations. This is mainly attributed to the insufficient analysis of the SCCP’s actual operation behaviors and the incomplete summarization of the SCCP’s dynamic-operation rules, which leads to significant obstacles in realizing the sequence-casting constraints of the simulation model when dealing with the variable processing time.

Based on the comprehensive observation of actual production behavior, dynamic operating rules for production-process adjustments were designed in this paper. Dynamic control of the simulation process was achieved using the built-in SimTalk language of Plant Simulation, thus avoiding the idealized treatment of the sequence-casting constraint evident in previous studies in the variable processing times. This paper focuses on the production processes of real steelmaking plants in China. A Plant Simulation software-based dynamic simulation model of the SCCP system is developed, and the effectiveness of the proposed model is validated by comparing it with the actual situation of the studied steelmaking plants. Currently, there are limited reports on the accurate simulation of real dynamic production processes in steelmaking plants based on simulation methods. In Section 2 of this paper, the simulation tools and corresponding simulation problems are described in detail. Additionally, Section 3 provides a detailed description of the process for building the simulation model. Moreover, Section 4 presents the experimental cases and evaluation indicators for the proposed model. Section 5 discusses and analyzes the experimental results. Finally, in Section 6, a summary of the paper is presented. 

## 2. Simulation Software Overview and Problem Description

### 2.1. Introduction to Simulation Software

The digital factory is an inevitable trend for future industrial development [27,28]. The Plant Simulation software is one of the powerful tools available for building a digital factory, one which can comprehensively consider all the production elements of the SCCP, and then build a digital factory that can accurately simulate the actual operating conditions. Compared to other research methods, the production-simulation approach is more closely aligned with actual operations, which facilitates quantitative analysis and verification of actual production results. As a result, it has been widely applied in the study of SCCP production coordination optimization [29,30,31]. 

Plant Simulation provides a rich set of graphical functional modules which can be categorized into logistics, resources, information, user interface, and data analysis modules, among others. These modules allow users to create virtual workstations corresponding to the processes in a real factory. Through the module settings, it is possible to simulate the basic operating conditions of the processes, including the control of parts’ entry and exit, process processing time, processing quantity, and processing time statistics. Additionally, Plant Simulation integrates the SimTalk programming language with a wealth of control attributes, which enhances the simulation flexibility of Plant Simulation. This flexibility is particularly advantageous in handling the complex logical relationships among different processes within the production, allowing different simulation demands to be precisely addressed. It is essential for implementing dynamic-operation rules and simulating dynamic production processes.

In the process of modeling based on Plant Simulation, the most commonly used controls include Sources, Drains, Stations, AssemblyStations, DismantleStations, Buffers, etc. Table 1 provides a functional introduction to the commonly used controls in the simulation modeling. Each control is equipped with a variety of configurable parameters and can bind the “Method” program to achieve flexible control. At the same time, each control offers multiple attributes, including the current status of the control, the import and export state, and the current material information, which are used for the development of Method-control programs. This allows the controls to closely match the actual objects as much as possible, achieving precise simulation modeling.

### 2.2. Description of Problem and Relevant Assumptions

**Figure 1:**
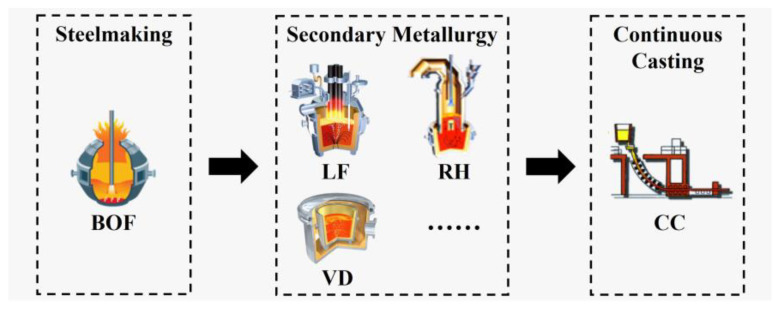
Process-route of quality steel manufacture [33].

The steel manufacturing process is an open, far-from-equilibrious, irreversible, and complex process system integrated by non-linear coupling of unit processes with different structure-functions [4]. In contrast to discrete manufacturing processes, the most typical characteristic of SCCP is the uninterrupted casting in the CC process, which is driven by extremely important production-cost considerations. The quality steel manufacture process is shown in Figure 1. The high-temperature production process ranges from 1500–1700 °C, which, along with the inclusion of high-temperature phase transitions, serves as another distinctive characteristic that distinguishes SCCP from other manufacturing processes. Based on these specialized production characteristics, SCCP generates a series of production constraints which can be primarily categorized as follows:

(1) There are strict time constraints on the process connection, as the delivery of qualified molten steel to the continuous caster needs to be punctual in order to ensure the continuous, uninterrupted casting of adjacent heats on the same caster.
(2) The smelting process of each heat has strict constraints on its the processing sequence; each heat needs to undergo subsequent smelting only after the completion of the preceding smelting process.
(3) The inter-process transfer is subject to strict timeliness constraints in which high-temperature molten steel needs to be delivered to the subsequent smelting process within the limited timeframe to comply with the steel temperature control protocols.
(4) The processing capacity of each device is subject to strict quantitative constraints, as the same device can only commence the subsequent heat after the completion of the preceding heat.

The aforementioned constraints fully reflect the complexity of SCCP, and the simulation process needs to be skillfully designed to meet the above constraints. Furthermore, the simulation modeling process also needs to deal with other factors that will affect the simulation accuracy, such as weight of heat, processing time, device stability, and so on. In the related literature [23,24,25,26,29,30,31], the above factors have been idealized, including the use of constant processing times, constant weights of heats, and unchanged casting speeds. These idealized assumptions will drive the simulation model to deviate from the real world, affecting the accuracy of simulation modeling. For the purpose of simulating the SCCP dynamic production process more accurately, the idealization of the aforementioned factors is avoided in this paper, and instead, real-time generation is employed to dynamically set these parameters based on statistical results from on-site data. The following are the basic assumptions of this paper prior to the simulation:

(1) The schedules of the production plans to be used in the simulation are known, including, but not limited to, the expected starting (completion) time of each cast, number of heats in each cast, the target-continuous caster, the device assignments for each heat, and the production steel grades and target casting speed.
(2) The molten-steel temperature meets the requirements of the production process and is not affected by transportation.
(3) Casting breaks caused by, e.g., device failures or order changes are not considered.
(4) The ladle-overhaul cycle is approximately 80 times, and the case of ladle overhaul is not considered in this paper.
(5) The on-site production environment and control level are consistent with the characteristics of the smelting cycles and weights of heats in previous processes, values which can be determined based on historical statistical data.

## 3. Simulation Modeling

The simulation modelling process can be mainly divided into three steps. Step 1 is the statistical analysis of the on-site data used as the driving data for the simulation model, including heat weight, CC casting speed range, processing cycle for each process, overhead crane transfer time among adjacent processes, ladle turnover cycle, etc. Step 2 establishes the static model of the production site according to the workshop layout of the target steelmaking plants, a process which mainly includes determining the location of each device and placing them, and then establishing the physical-connection relationships among the devices and generating movable objects such as the molten steel and ladle. Step 3 involves the design of the dynamic control program for each device and ladle based on SimTalk language and realizes the simulation of the production process through the dynamic judgment and decision-making for the real-time production-operation situation.

### 3.1. Drive Data

The steelmaking process is a complex and multi-factorial system production process, subject to factors such as unclear metallurgical mechanisms and frequent disturbances. The processing time of each smelting process is not constant. Therefore, with the aim of achieving an accurate simulation of the dynamic operational conditions, it is necessary to generate accurate statistical analyses and descriptions of the actual on-site operational data before building the simulation model in order to obtain the driving data for the simulation model.

As the first process in the SCCP, the converter is subject to intense physical–chemical reactions in completing the transformation from hot metal to molten steel, a process with complex metallurgical mechanisms and numerous factors causing disturbances. Consequently, the converter smelting cycle is relatively uncontrollable. The fluctuations of the converter smelting cycle, especially the continuous tapping delay, undoubtedly cause great damage to the stable operation of the entire production process and are often a crucial factor in interruptions in sequence casting. The flexible production characteristics of LF and RH refining contribute to their being regarded as typical buffering processes used for production-operation rhythm adjustment [34]. Hence, their refining cycles are relatively controllable, which is consistent with the results of on-site surveys. In this paper, the distribution of converter smelting cycle is analyzed, and the standard processing cycles and buffering time for the refining processes are examined. Additionally, due to the constraints of steel grade solidification characteristics and the blank quality requirements, the allowable casting speeds for different blank cross-section sizes are specified by the process specifications. Under the condition of fixed cross-section size in CC, there will be a strong positive correlation between the heat weight and the casting cycle. Therefore, a detailed statistical analysis of the heat weight is also performed for the simulation modeling.

**Figure 2:**
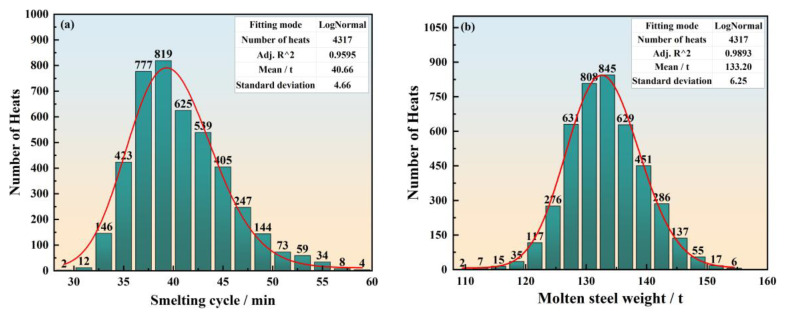
Statistical results of data analysis for (**a**) converter smelting cycle and (**b**) molten-steel weight.

The 4317 sets of historical data are taken from the studied steelmaking plants during the period of October to December in 2021. Figure 2 shows the distributions of the converter smelting cycle and heat weight in the studied steelmaking plants. From Figure 2a, it can be observed that the distribution of the converter smelting cycle has a left-skewed peak and a rightward extending long tail, indicating a positively skewed distribution. The reason behind this skewed distribution characteristic is the frequent occurrence of disturbance events that disrupt the timely tapping of the converter, which in turn leads to the tendency of the converter smelting cycle to extend in the direction of increase. Skewness coefficient is a statistical parameter that reflects the degree and direction of asymmetry in data distribution. The larger the absolute value of the skewness coefficient is, the more serious the skewness of the distribution is.

The converter smelting cycle distribution has a skewness coefficient of 0.73, indicating a skewed distribution. Therefore, the commonly used normal distribution, as seen in related literature [23,24], is not entirely suitable for describing the distribution of the converter smelting cycle. Instead, the lognormal distribution, with its more upwardly fluctuating values, is better suited for describing datasets with positively skewed distributions. This conclusion is further supported by the good-fit measures. For the converter smelting cycle, the lognormal distribution has a good fit of 0.9595, which is significantly superior to the normal distribution’s good fit of 0.9318. Similarly, as depicted in Figure 2b, steelmaking plants tend to increase the heat weight to enhance steel production in the real-world production environment. As a result, the heat weight also follows a positively skewed distribution, with a skewness coefficient of 0.15, indicating a slight skewness. In terms of good fit, the lognormal distribution displays superior good fit, with a value of 0.9893, in contrast to 0.9839 for the normal distribution.

Based on the data analysis and on-site investigation, the statistical results for the processing cycles, soft-blowing times and buffer times of refining processes in the studied steelmaking plants are presented in Table 2, in which soft blowing is an integral part of the refining process. As for soft blowing, its primary role is to further improve the quality of the molten steel, and its secondary role is to ensure continuous production. Therefore, with the objective of ensuring the lowest time value, the soft blowing can be compressed. This process characteristic makes the soft-blowing process undertake the main buffering task. In accord with the results of an on-site investigation and data analysis, a buffer time of 5 min was set in the soft-blowing process for the studied steelmaking plants. Table 3 displays the allowable casting speed ranges for different section sizes in CC. Accordingly, these statistics were utilized to define the processing cycles for each process in the simulation modeling.

In addition, the simulation process also involves the transportation of ladles among different processes. The actual transfer time among different processes is not constant. Therefore, in the context of the studied steelmaking plants, and based on their actual production situations, the model has magnified the transfer time, and the ladle transfer time among the different processes is set to a constant value of 12 min, which is longer than the shortest transfer time among the different processes.

### 3.2. Static Modeling

**Figure 3:**
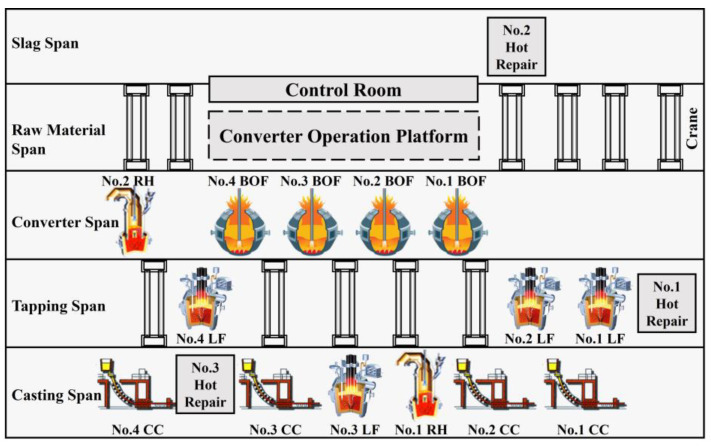
Workshop layout of studied steelmaking plants.

After the simulation-driven data is obtained, a static model of the simulation objects is built, based on the workshop layouts of the studied steelmaking plants, as shown in Figure 3. The steelmaking plants under study include four converters (No. 1 BOF to No. 4 BOF), four LF refining furnaces (No. 1 LF to No. 4 LF), two RH refining furnaces (No. 1 RH and No. 2 RH), and four continuous casting machines (No. 1 CC to No. 4 CC). Additionally, the steelmaking plants also have three ladle hot-repair stations (No. 1 HotRepair to No. 3 HotRepair) and several overhead cranes, five of which are positioned within the tapping span.

**Figure 4:**
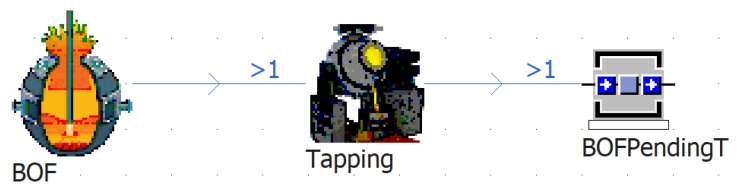
Functional decomposition of production process in BOF.

**Figure 5:**
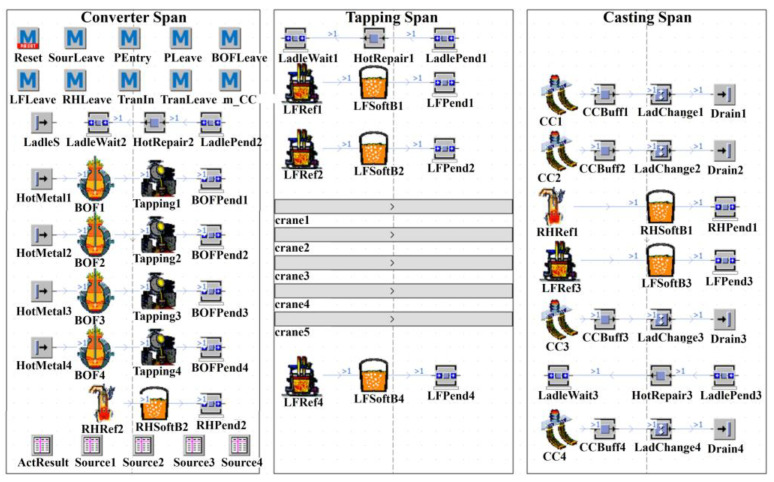
SCCP simulation model created using Plant Simulation.

Given the on-site positions of each device, the simulation workstations can be arranged reasonably within the simulation platform. The various devices in the physical workshop are mainly distributed in the converter span, tapping span, and casting span. Therefore, when building the static architecture of the simulation model, the focus is on the three operational spans of the steelmaking plants. The three operational spans, named Converter Span, Tapping Span, and Casting Span, are first created within the simulation platform; then, the corresponding simulation stations are added to the simulation span according to the device configuration of each operational span in the physical workshop. It is important to note that, since each process is a collection of multiple operations, it is advisable to decompose the process functions into multiple simulation workstations during the simulation modeling process in order to enhance clarity and intuitiveness. Taking the BOF as an example, the decomposition of BOF is shown schematically in Figure 4. The BOF was decomposed into a processing workstation, an assembly workstation, and a buffer workstation, representing the smelting process, molten-steel tapping process, and liquid-steel waiting process, respectively. The other processes are also decomposed according to their functions. Finally, the static architecture of the multi-process dynamic-operation simulation model for SCCP is gradually established, as shown in Figure 5. The introductions and functions of the main simulation objects in the static model are summarized in Table 4.

In addition to the above logistics objects, the simulation model also includes three types of movable objects, namely, parts, containers, and carts, representing molten steel, ladles, and overhead cranes, respectively. All three types of movable objects offer customizable attribute settings, including the current state of the movable objects, import and export status, current controls information, etc., which can be used for the development of Method control programs, which can in turn be flexibly configured based on simulation requirements to facilitate the implementation of various simulation-based functionalities.

### 3.3. Dynamic Modeling

To satisfy the sequence-casting constraint, it is essential to develop dynamic-operation rules for SCCP based on the static model. This is because the SCCP is subject to dynamic variations, and the schedules that can initially meet the sequence-casting constraint with excellent performance may become ineffective during actual production due to fluctuations in the processing cycles of the preceding processes. Therefore, the key to achieving dynamic simulation lies in dynamic-operation rules implemented through SimTalk.

(1) Dynamic-operation rules

Dynamic-operation rules are implemented to provide guidance for schedules, enabling real-time adjustments that ensure the stability of the entire production process when normal production is disrupted. These rules primarily encompass refining buffer adjustments and continuous casting controls. The specific operational rules are outlined as follows:

Rule 1: When there is a delay in the completion time of converter smelting, and this delay is within the maximum buffer time allowed for the refining process, the start time and duration of the refining process are adjusted based on the converter delay time.

Rule 2: If there is a delay in the completion time of converter smelting, and the delay time exceeds the maximum buffer time allowed for the refining process but is still within the adjustable time of the CC, the CC casting speed is promptly reduced based on the converter delay time in order to extend the casting completion time of preceding heats, and the start time and duration of the refining process are adjusted according to the maximum refining buffer capacity.

Rule 3: If there is a delay in the completion time of converter smelting, and the delay time exceeds the sum of the maximum buffer time allowed for the refining process and the adjustable time of the CC, there will be a temporary interchange of two converter smelting heats; the other converter smelting heats are urgently called to supply the strand, and the start time and duration of the refining process as well as the CC casting speed are accordingly adjusted.

Rule 4: When the completion time of the refining heat is delayed, and the delay time is within the maximum buffer time allowed for the refining process, the refining duration is adjusted based on the delay time.

Rule 5: When the completion time of the refining heat is delayed and the delay time exceeds the maximum buffer time allowed for the refining process but is within the CC adjustable time, the refining duration is accordingly adjusted, and the casting speed is immediately reduced.

Rule 6: If the completion time of the refining heat is delayed and the delay time exceeds the sum of the maximum buffer time allowed for the refining process and the CC adjustable time, the steel grade of the other refining device is urgently reordered to supply the strand, and the refining duration and the CC casting speed are adjusted accordingly. In this scenario, there will be a temporary interchange of heats between the two refining devices.

Rule 7: Empty ladles, after completion of casting, are prioritized for repair at the hot-repair station to ensure fewer waiting ladles. If multiple hot-repair stations have the same number of ladles waiting, the ladle is sent to the nearest hot-repair station.

Rule 8: The ladle with the longest waiting time is given priority to receive the molten steel when converter-tapping. If there are multiple ladles with the same waiting time, the ladle nearest to the converter is chosen.

**Figure A1:**
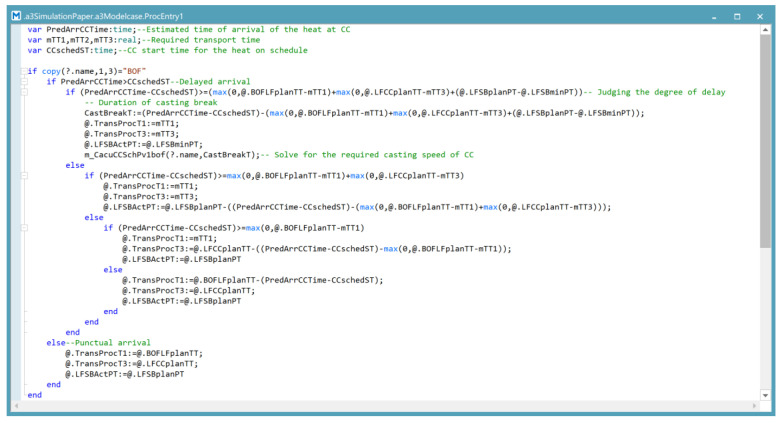
Part of the SimTalk program for dynamic-operation control within converter.

**Figure A2:**
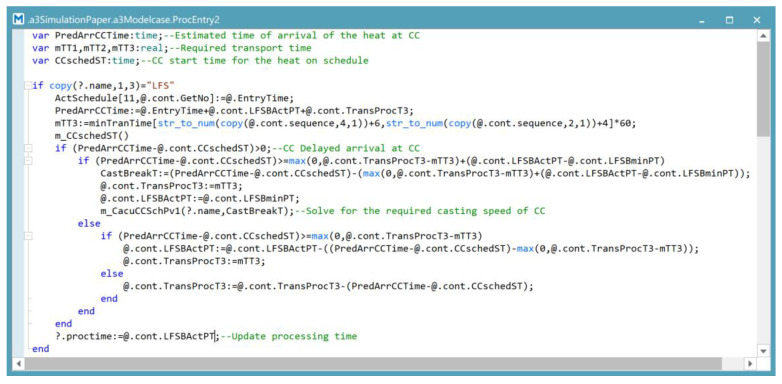
Part of the SimTalk program for dynamic-operation control within refining.

Based on the above rules, the corresponding control program can be written for Method control through the SimTalk programming language, as shown in Figure A1 and Figure A2 in Appendix A, which illustrates an example of a control program written in SimTalk. After encapsulating the Method control, it is attached to the corresponding workstation. This allows the program segment to be triggered when a movable object reaches this workstation, enabling the appropriate control actions.

(2) Dynamic operational programs

**Figure 6:**
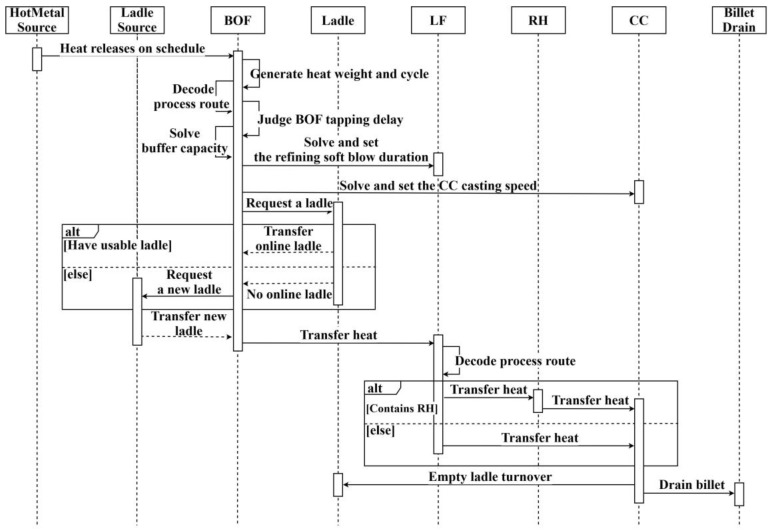
Logic control mechanism of SCCP simulation model.

The logic control mechanism of the SCCP simulation model is illustrated in Figure 6. The control programs for each workstation are encapsulated within their respective Method objects, and the corresponding Methods are triggered when movable objects are conveyed to the respective workstations. When elaborated step by step, the operation control of each process in the simulation process can be better illustrated.

**Figure 7:**
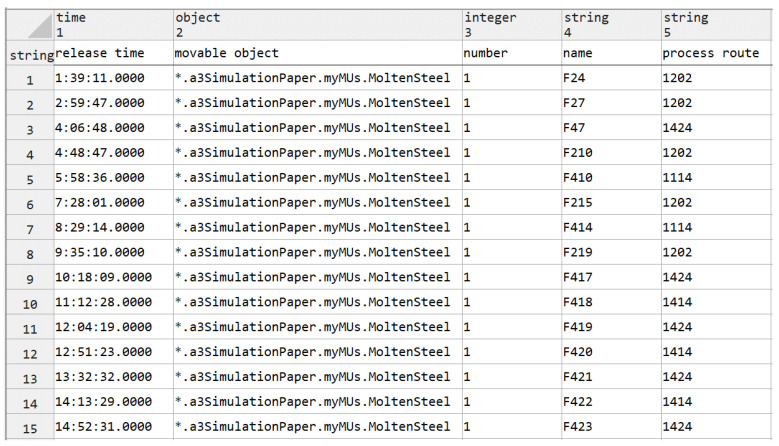
Example of heats production information sheet. (* is file path of movable object in shorthand)

**Figure 8:**
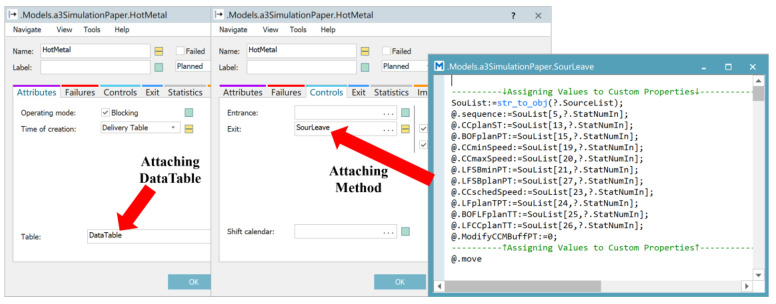
Attaching DataTable and Method for HotMetal.

In Step 1, set up the simulation data and release the heats according to the schedule scheme. Create a DataTable, as shown in Figure 7, for storing the schedule scheme used in the simulation experiment, including the release time, process route, heat name, and other information associated with each heat. Bind the DataTable to the corresponding HotMetal (Source), as shown in Figure 8. At the same time, write a Method for the initialization of the customized attributes of each heat.

**Figure 9:**
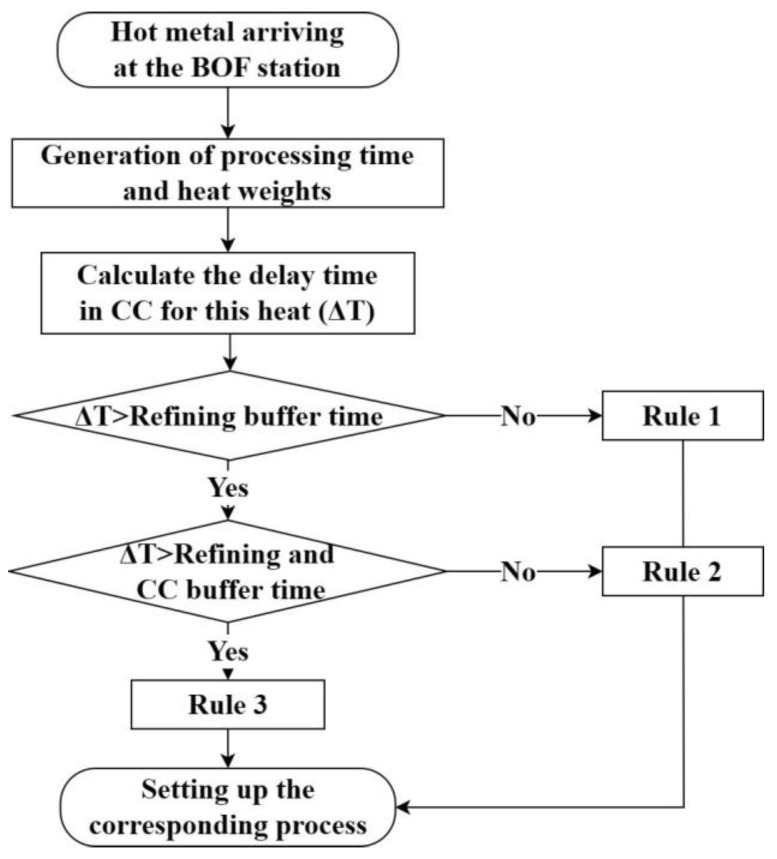
Determination of dynamic-operation rules in converter.

In Step 2, generate the processing time and heat weight for each heat according to the data distribution characteristics and determine the dynamic-operation rules. When the HotMetal releases iron to the converter, the converter will generate the corresponding heat-weight and processing time for each heat and assess the current production situation to determine the required dynamic-operation rules at the time of molten-steel-tapping. The corresponding judgment process is shown in Figure 9, and the subsequent processes are set according to the rules. This step is the key to realizing the caster sequence-casting target. Previous studies focused on setting corresponding processing times for each process, lacking the assessment of current production-operation situation and the selection of dynamic-operation rules. This lack of dynamic assessment and adjustment in the production process has, in related studies, led to the dilemma involved in attempting to meet the sequence-casting constraint. By incorporating dynamic operating-rule assessment and selection into the simulation process, the gaps identified in previous studies can be effectively addressed.

**Figure 10:**
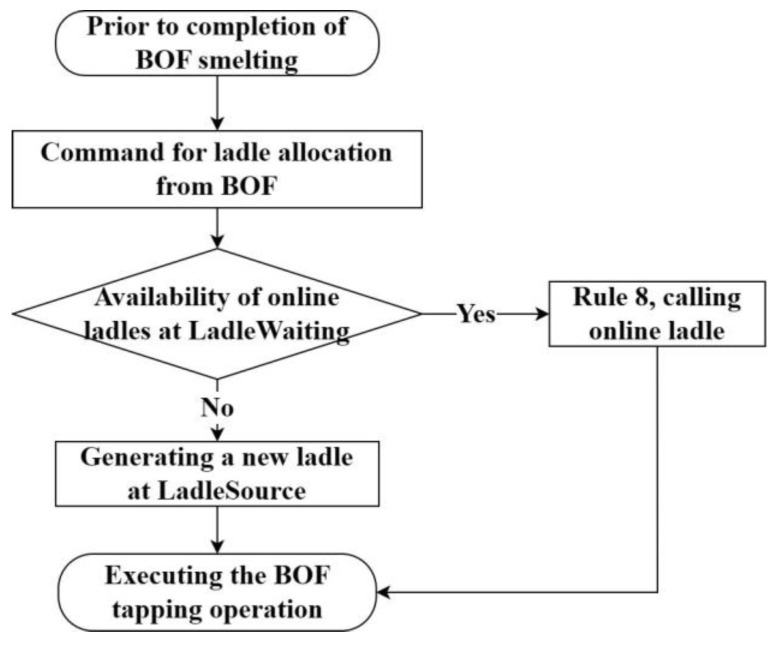
Process of assigning ladles to heats.

Step 3 is the ladle allocation for converter-tapping. Before the converter completes the smelting, the converter sends out a ladle allocation command. At this point, firstly, determine whether there is an online ladle available in the LadleWait station, and secondly, choose to call a new ladle from LadleS if there is no ladle online. The ladle allocation process is shown in Figure 10. After the ladle arrives at the converter-tapping station, the tapping operation is performed. The corresponding simulation process is the combination process of the molten steel and the ladle. After passing through this station, both will be composed of a new movable object.

**Figure 11:**
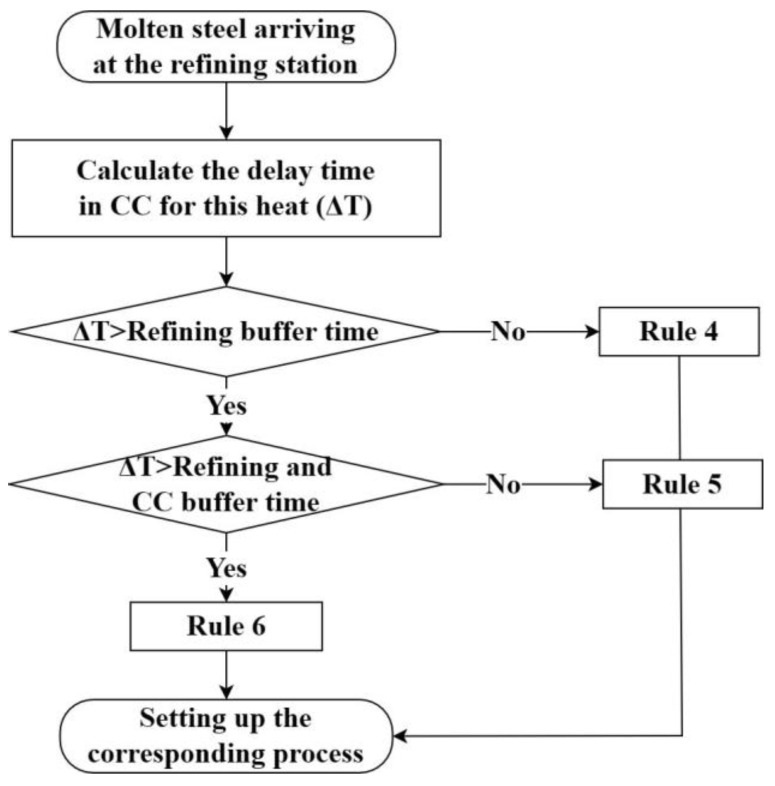
Determination of dynamic-operation rules in LF refining.

Step 4 Implements the judgement of the dynamic-operation rules for the refining processes. After the ladle allocation and molten-steel-tapping at the converter, the ladle with molten-steel is sent to the corresponding refining process according to the designed process route. The refining process will perform the refining according to the processing time set by the previous dynamic-operation rules and determine the required dynamic-operation rules at that time. The corresponding judgment flow is shown in Figure 11. After the LF refining is completed, the molten steel is sent to RH refining or the CC process for casting according to the requirements of the process route.

Step 5 involves the separation of molten steel and ladle in the CC and the determination of the empty ladle’s destination. After the molten steel arrives at the CC process, it is cast at the casting speed set by the previous rules, and the molten steel separates from the ladle at the LadChange station. The separated molten steel is recovered by the Drain, and the empty ladle will then return to the HotRepair station for hot repair according to Rule 7.

## 4. Simulation Experiment and Model Evaluation

### 4.1. Simulation Instances

The reality of the studied steelmaking plants is that when all four continuous casters are running, the production capacity of the four converters is not sufficient to meet the molten-steel demand of the casters, making it difficult to maintain the continuous operation of the steelmaking plants. Due to the productivity-matching issue among the BOFs and CCs in the studied steelmaking plants, the “4BOF-3CC” production mode has emerged as a suitable option [11]. The “4BOF-3CC” mode refers to four converters supplying molten steel to three casters, which is the most frequently utilized production-operation mode in the studied steelmaking plants. Four simulation instances based on actual production are chosen in this paper, as shown in Table 5. Each instance corresponds to a shutdown of a specific continuous caster, namely No. 1 CC, No. 2 CC, No. 3 CC, and No. 4 CC, respectively. Given that more than 80% of the total production yield in the studied steelmaking plants is carried out under the “4BOF-3CC” mode, the designed simulation instances in this paper effectively capture the actual production scenarios and possess strong representativeness. The joint validation of these four instances can more comprehensively reflect the accuracy of the simulation model in simulating actual production processes.

### 4.2. Evaluation Indicators

In accordance with the analysis in Section 2.2, the following evaluation indicators were selected for this paper: the total completion time of the production plan, the transfer time of heat among different processes, and the ladle turnover frequency. The specific meanings and calculation formulas for these three evaluation indicators are described in detail in the following:

The total completion time ($T_{\mathrm{Total}}$, min) represents the elapsed time of each simulation instance. It serves as a metric used to evaluate the overall fidelity of the simulation model by comparing the simulation results with real-world results. Equation (1) illustrates the calculation of the total completion time.

(1) $$T_{\mathrm{Total}}=\tau_{\mathrm{lastH},\mathrm{CC}}-\tau_{\mathrm{firstH},\mathrm{BOF}}$$

where $\tau_{\mathrm{firstH},\mathrm{BOF}}$ is the converter start time of the first heat in the schedule, and $\tau_{\mathrm{lastH},\mathrm{CC}}$ is the casting completion time of the last heat in the schedule.

The transfer time of the heat ($T_{\mathrm{MTrans},P1\to P2}$, min) is the average time needed for heat transport from the preceding smelting process to the following ones, covering both waiting and transport time. The calculation formula for the transfer time of the heat is given by Equation (2). This indicator can be used to evaluate in detail the similarity between the simulation model and the actual production.

(2) $$T_{\mathrm{MTrans},P1\to P2}=\frac{\sum_{i=1}^{n}\tau_{P2,\mathrm{arrival}}-\tau_{P1,\mathrm{finish}}}{n}$$

where $\tau_{P1,\mathrm{finish}}$ is the smelting completion time in the previous process; $\tau_{P2,\mathrm{arrival}}$ is the time of the molten steel arriving at the subsequent process; and n is the total number of heats in the schedule.

The frequency of ladle turnover ($N_{\mathrm{MTurn}}$) represents the average number of times each ladle is utilized throughout the entire simulation process. It can be calculated using Equation (3). Ladles are essential components of the production organization in the steelmaking plants, serving as crucial auxiliary equipment, and ensuring the continuous and stable operation of production. This indicator provides a valuable assessment of the fidelity of the simulation model in terms of the auxiliary resources of production.

(3) $$N_{\mathrm{MTurn}}=\frac{\sum_{j=1}^{m}N_{j}}{m}$$

where $N_{j}$ is the number of times each ladle was used, and m is the number of ladles used in production.

The three indicators mentioned above provide a comprehensive assessment of the simulation model’s effectiveness in emulating real-world scenarios, taking into account the overall performance, local processes, and utilization of the auxiliary resources of production. As a result, these indicators are appropriate for evaluating the validity of the simulation model.

## 5. Analysis of Results, and Discussion

The dynamic simulation results (referred to as C1) based on the instances discussed in Section 4.1 will be analyzed in detail in this section. Additionally, the standard results (referred to as C2) obtained by executing the standard processing time will also be compared. Finally, the actual on-site operational results (referred to as C3) have been recorded for the purpose of comparison. The comparisons among C1, C2, and C3 will provide insights into the effectiveness and the fidelity of the simulation model in emulating the real production process.

**Figure 12:**
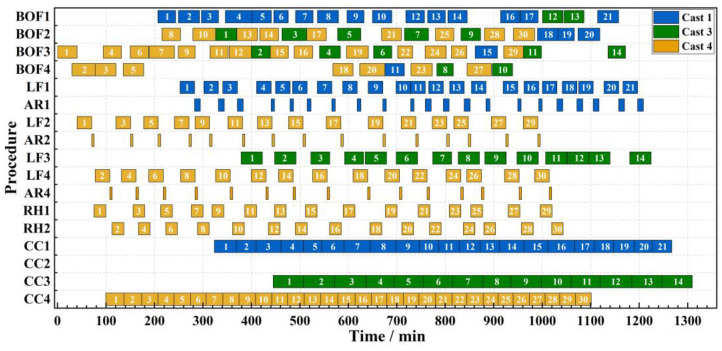
Gantt chart for Instance 2.

The sequence-casting constraint in CC is a critical constraint in real production processes and serves as a vital evaluation metric for simulation models. In the proposed simulation model, the integration of dynamic-operation rules enables the production process to promptly adjust the processing cycles of each heat within the process constraints, based on real-time production conditions. Consequently, sequence casting in the caster is realized in all four instances. Taking Instance 2 as an example, the simulation production process can be visually represented by the Gantt chart shown in Figure 12. The vertical axis shows the various processes of the SCCP, within which AR is the soft-blowing process of LF refining, and the horizontal axis shows the timeline of the production process. Each small rectangle in the chart represents a heat, with different colors indicating different casts, and the numbers within the rectangles represent the sequence of the heats in the cast. It can be observed from the Gantt chart that all casters successfully satisfy the sequence-casting constraint.

**Figure 13:**
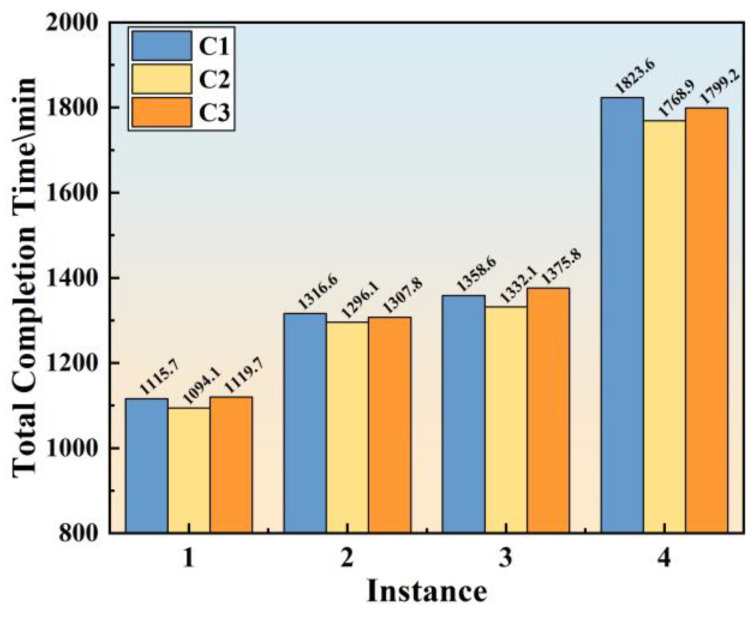
Comparison of total completion times of production plans for four instances.

Figure 13 presents a comparison of the total completion times of the production plan for the four instances, from which it can be observed that the total completion times of C1, C2, and C3 are relatively similar among the instances, with C2 having a slightly shorter total completion time than C1 and C3. This is due to the fact that C2 represents a simulation result under standard-processing-cycle conditions, so the production process does not lead to a reduction in the casting speed of the caster. The caster casts according to the predetermined casting speed, and the total completion time is not extended. The differences between C1 and C3 are 4.0 min, 8.8 min, 17.2 min, and 24.4 min, respectively, with corresponding deviation rates of 0.36%, 0.67%, 1.25%, and 1.36%. Although the simulation model exhibits a discrepancy of less than 3%, such a level of deviation is inevitable and acceptable in a simulation experiment on such a large scale [24,29]. This indicator implies that the simulation model can ensure similarity with the real physical system within a global perspective.

In addition to assessing the overall similarity, analyzing the local simulation results of the simulation model and the utilization results for auxiliary resources of production can provide further validation of the fidelity of the simulation model. Table 6 presents a comparison of the transfer time of the heat between BOF-LF, LF-CC, and RH-CC, along with the ladle utilization data for the four instances. This comparison allows an evaluation of the simulation model as to its emulation of the heat transfer processes and auxiliary resource utilization in the actual production environment.

From Table 6, it can be observed that there are some variations in the transfer times for the heat between adjacent process in C1, C3, and C2. The inter-process transfer time of the heat for C2 is greater than those for C1 and C3. In contrast, C1 and C3 represent dynamic operational results under fluctuating processing cycles, resulting in compressed inter-process transfer times for the heat. The inter-process transfer times of the heat for C1 and C3 are relatively similar, and the maximum difference in all instances was less than 1 min, occurring in the LF-CC transfer process of Instance 4; the other transfer-time differences are all less than 0.8 min, which verifies the fidelity of the simulation model. In addition, C1, C2, and C3 show the same results for all four instances in terms of the numbers for ladle usage and turnover frequency. This consistency in the indicators of auxiliary resources of production further validates the accuracy of the simulation model.

According to the comparisons of the above indicators, it can be seen that the main difference in C2 relative to C1 and C3 lies in the transfer times of the heats among the processes. Since C2 does not include the fluctuations in the processing times, each heat can be smelted on time according to the schedule without the need to reduce the heat transfer time, as in actual production. Therefore, the heat transfer time in C2 is closer to the planned value in the initial schedule, deviating from the actual operational results in C3. For C1 and C3, the indicators are close to each other, with only minor deviations. It is not difficult to understand the reasons for the minor deviations. C1 generates the processing time for each heat according to the distribution characteristics, and a set of heat processing times produced in this way will not correspond one-to-one with the set of processing times in the actual production results. As a result, there will be certain deviations at some stages of the simulation process compared to the actual production process. However, for the overall statistical results of the simulation, this deviation is controllable and acceptable. In addition, the simulation model assumes that only the disturbance of processing time fluctuation occurs, but in actual production, there are other disturbances that affect the production process, leading to variations in evaluation indicators such as the total completion time and the transfer times of heats. This is also one of the reasons for the differences between simulation and actual results. The above comparison demonstrates the reliability and fidelity of the simulation model and indicates its ability to emulate real production processes effectively and accurately. However, it should be further clarified that the model in this paper has certain limitations when addressing simulation experiments on a larger scale. The model assumes that the molten-steel temperature meets the production specifications and does not take into account the ladle overhaul situation. In the case of larger simulation experiments involving more heats, the impacts of these assumptions will be magnified, affecting the accuracy of the simulation model.

This model allows the study of the on-site applicability of different schedules. Moreover, it is possible to investigate the operational efficiency and robustness of different schedules. Two schedules were selected for comparison: the heuristic algorithm-based schedule (C4) from reference [35], and the heuristic algorithm with the “furnace–machine coordinating” mode optimization-based schedule (C5) from reference [11]. The difference between the two schedules is that C5 incorporates the “furnace–machine coordinating” mode for device assignment during the solution-finding process. The operational efficiency and robustness of the two schemes are evaluated by comparing the total completion time of the production plan (*T*_Total_), transfer waiting time of heats (*T*_Waiting_, which is the heat transfer time minus the required transfer time), and the number of rescheduling times (*N*_re_) in 10 repeated experiments under dynamic conditions.

In Table 7, comparisons of the simulation results for each schedule are presented. Here, it can be observed that the *T*_Total_ and *T*_Waiting_ of the C4 and C5 have different magnitudes under different simulation instances. Therefore, to better evaluate the two schedules, the weighted evaluation values of each evaluation indicator are calculated based on each instance’s weight value of production yield in the studied steelmaking plants. The calculation formula is shown in Equation (4).

(4) $$E_{y}=\sum_{\alpha =1}^{4}\begin{array}{cc} \omega_{\alpha }e_{\alpha ,x}, & x=4,5 \end{array}$$

where *E_y_* is the weighted evaluation value of a certain evaluation indicator, and y represents *T*_Total_ or *T*_Waiting_; *ω_α_* is the weight value corresponding to the instance α, which is determined by the actual production yield proportion of each instance, and for the four instances in Table 5, the weight values are 0.15, 0.10, 0.65, 0.10, respectively; and *e_α_*_,4_ and *e_α_*_,5_ are the evaluation indicator values for C4 and C5 under instance α, respectively.

The weighted evaluation values of *T*_Total_ and *T*_Waiting_ for the two schedules can be obtained from Equation (4). For C4, the weighted values of *T*_Total_ and *T*_Waiting_ are 1363.6 min and 458.6 min, respectively, while for C5, the weighted values of *T*_Total_ and *T*_Waiting_ are 1354.9 min and 413.2 min, respectively. Compared to C4, C5 has a reduction of 8.7 min in the weighted total completion time of the production plan and a reduction of 45.5 min in the weighted transfer waiting time of heats. C5 demonstrates superior operational efficiency, which is attributable to the introduction of the “furnace–machine coordinating” mode in the schedule. The optimization based on the “furnace–machine coordinating” mode is derived from empirical knowledge summarized from on-site production. The optimized schedule reduces the randomness of process/device assignment, and the clear correspondence among devices ensures the smooth operation of the material flow, consequently reducing both the total completion time and transfer waiting time in the actual environment.

The number of rescheduling times in a dynamic environment can reflect the usability of a schedule under the actual conditions. Fewer rescheduling times indicates a stronger robustness of the schedule, which is beneficial for enhancing the adaptability of the schedule in the field. From Table 7, it can be seen that for the *N*_re_, the rescheduling times of C5 in all instances are not larger than those of C4, which indicates that the C5 has better field applicability. Due to the absence of the “furnace–machine coordinating” mode in C4, there is no obvious correspondence among the devices, resulting in larger transfer distances among devices, compared to C5. This leads to increases in the required transfer times for C5. This change reduces the transfer compressible time and decreases the buffering capacity during production, which leads to an increase in the number of rescheduling times. Through the above analysis, it can be seen that schedule C5 is superior to C4, and the optimization effect of the “furnace–machine coordinating” mode on the availability of the schedule is verified based on the dynamic-operation simulation model proposed in this paper.

## 6. Conclusions

The conventional methods for analyzing production-operation data have limitations when it comes to providing detailed analysis and emulation of large-scale dynamic production processes. In contrast, production-simulation research offers distinct advantages by comprehensively considering multiple factors that influence production. It has the capability to address complex production issues with high accuracy and at a low cost. This makes it an important approach for analyzing the adaptability of various production-operation optimization strategies in the field. In response to the SCCP with typical process manufacturing features, the designs and detailed descriptions of certain SCCP dynamic operational rules are presented in this paper. Additionally, a dynamic operational control program is developed using Plant Simulation software, and a dynamic simulation model is established for the SCCP. By allowing for the reasonable adjustment of the processing cycle of each heat based on real-time production conditions and within production-process constraints, the simulation model enables the continuous operation of casters in the SCCP within a discrete simulation platform. The proposed dynamic simulation model is validated by comparisons with real steelmaking plants. Evaluation indicators, including the total completion time of the production plan, transfer time of the heat among processes, and ladle turnover frequency, are employed to assess the proposed dynamic simulation model. The above indicators show a strong consistency between the simulation results and the actual results. This confirms the effectiveness and validity of the proposed dynamic simulation model presented in this paper.

Based on the confirmed effectiveness of the simulation model, the model was used for the comparative validation of two schedules. The results show that the schedule based on the “furnace–machine coordinating” optimization has better operational effectiveness. After the optimization of the “furnace–machine coordinating” mode, the weighted value of total completion time of the production plan is reduced by 8.7 min, and the weighted value of transfer waiting time of heats is reduced by 45.5 min, while the number of rescheduling times is reduced, which verifies the optimization effect of the “furnace–machine coordinating” mode as to the schedule. Finally, in order to address the limitations from the modeling assumptions, we will further investigate the impacts of molten-steel temperature variations and ladle overhaul cycles as well as the integration of the continuous casting simulator from Ref. [22] in order to develop a more accurate simulation model of the SCCP in subsequent studies.

## Figures and Tables

**Table 1:** Common controls within Plant Simulation [32].

Controls	Function
Sources	Used for the generation of raw materials, creating the objects that will be processed in the subsequent production line. The manner and rate of material generation, as well as the distribution followed, can be set in its “Options” tab.
Drain	Used for material recycling. Once the material reaches the Drain, it signifies that the material has completed all production processes.
Station	Used for the processing of materials, representing a single processing workstation. It allows for the setting of processing time, setup time, repair time, average downtime, and other information, in order to closely align with actual conditions.
Assembly	Used for the assembly of materials, combining multiple materials to form a movable object.
Dismantle	Used for the disassembly of materials, breaking down a single material into multiple movable objects.
Buffer	Used as an area or container for the temporary storage of materials
Connector	Used to connect different controls, forming a continuous material flow route.
Line	Used for transporting materials, and has an actual length. The corresponding speed can be set.
Transporter	Used to carry materials and transport them to a designated location.
Track	Used to place Transporters and implement their movement.
DataTable	Used to store data and information which can then be bound to other controls for reading and writing data.
Method	Used for writing programs to control the simulation behavior of various controls.

**Table 2:** Standard processing cycle and buffer time for secondary metallurgy.

Process Route	BOF-LF-CC	BOF-LF-RH-CC	
	LF Refining	LF Refining	RH Refining
Standard Cycle/min	44	51	36
Soft Blowing/min	12	5	12
Buffer Time/min	5	0	5

**Table 3:** Strand section size and casting speed for CC.

Caster	Strand Section Size mm^2^	Standard Casting Speed m/min	Maximum Casting Speed m/min	Minimum Casting Speed m/min
No. 1 CC	575 × 450 × 110 ^a^	1.00	1.10	0.80
	750 × 370 × 90 ^a^	1.00	1.10	0.80
	1024 × 390 × 90 ^a^	0.85	0.90	0.70
No. 2 CC	175 × 980	1.30	1.35	1.20
	175 × 1030	1.30	1.35	1.10
	175 × 1270	1.25	1.35	1.10
No. 3 CC	175 × 1270	1.25	1.30	1.10
	175 × 1360	1.20	1.30	1.10
No. 4 CC	200 × 1800	1.25	1.30	1.10
	250 × 1800	1.15	1.20	1.00
	250 × 2400	1.05	1.10	1.00

Note: ^a^ is the web thickness, mm.

**Table 4:** Introduction to and functions of main simulation objects in static model.

Process	Simulation Object	Control Type	Introduction and Functions
Source	HotMetal	Source	Source of hot metal for release or the temporary storage of hot metal according to static schedules
	LadleS	Source	Source of ladles, releasing new ladles according to production demands
BOF	BOF	Station	BOF smelting station for simulating the complete BOF smelting process
	Tapping	Assembly	BOF tapping station for the assembly of molten steel to the ladle
	BOFPend	Buffer	BOF waiting station for simulating the waiting process after the steel-tapping
LF	LFRef	Station	LF refining station for simulating refining operations such as energized heating and desulfurization before soft blowing.
	LFSoftB	Station	LF soft-blowing station for simulating the soft-blowing operation
	LFPend	Buffer	LF waiting station for simulating the waiting process after the completion of refining
RH	RHRef	Station	RH refining station for simulating refining operations such as vacuum extraction and degassing before soft blowing
	RHSoftB	Station	RH soft-blowing station for simulating the soft-blowing operation
	RHPend	Buffer	RH waiting station for simulating the waiting process after the completion of refining
CC	CC	Station	CC station for simulating the casting process in a caster
	CCBuff	Station	CC compensation station for dynamic adjustment of the CC casting time
	LadChange	Dismantle	Ladle changing station for separation of molten steel from the ladle
Ladle	LadlePend	Buffer	Empty-ladle waiting station for decision-making as to the hot-repair station and waiting for transportation
	HotRepair	Station	Ladle hot-repair station for simulating the ladle hot-repair process
	LadleWait	Buffer	Ladle baking station for simulating the process of ladle waiting to receive molten steel after the completion of hot repair
Drain	Drain	Drain	Billet recycling, for the collection of finished casting billets
Control	DataTable	DataTable	Storage of static schedules and results
	Method	Method	Control program, written in the SimTalk language and used to control the simulation behavior of each process

**Table 5:** Production plan for simulation experiments.

Instance	Caster	Process Route	Section Size/mm^2^	Heats in Cast/Heats	Casting Cycle/min
1	No. 2	BOF–LF–CC	175 × 1270	30	31
	No. 3	BOF–LF–CC	175 × 1270	15	60
	No. 4	BOF−LF−RH−CC	200 × 1800	23	38
2	No. 1	BOF–LF–CC	575 × 450 × 110	21	42
	No. 3	BOF–LF–CC	175 × 1360	14	59
	No. 4	BOF−LF−RH−CC	250 × 1800	30	34
3	No. 1	BOF–LF–CC	575 × 450 × 110	26	42
	No. 2	BOF–LF–CC	175 × 1270	33	30
	No. 4	BOF−LF−RH−CC	250 × 2400	31	28
4	No. 1	BOF–LF–CC	750 × 370 × 90	28	46
	No. 2	BOF–LF–CC	175 × 980	31	37
	No. 3	BOF–LF–CC	175 × 1360	26	57

**Table 6:** Comparison of simulation results.

Instance	Result	$T_{\mathrm{MTrans},P1\to P2}$/min			Ladle Number	$N_{\mathrm{MTurn}}$
		BOF-LF	LF-CC	RH-CC		
1	C1	10.84	10.51	8.82	18	3.78
	C2	12.00	11.55	11.32	18	3.78
	C3	10.39	10.33	9.17	18	3.78
2	C1	8.78	9.72	9.62	17	3.82
	C2	12.00	12.13	10.09	17	3.82
	C3	9.06	8.56	10.03	17	3.82
3	C1	7.81	10.75	8.05	19	3.58
	C2	12.00	11.56	11.69	19	3.58
	C3	7.99	10.07	8.21	19	3.58
4	C1	9.81	12.04	-	15	4.53
	C2	12.00	12.03	-	15	4.53
	C3	9.23	13.01	-	15	4.53

**Table 7:** Simulation results as to comparisons of schedules.

Instance	Result	*T*_Total_/min	*T*_Waiting_/min	*N*_re_
1	C4	1109.9	438.7	4
	C5	1099.1	395.4	3
2	C4	1304.5	243.6	3
	C5	1310.3	223.7	3
3	C4	1365.9	535.4	5
	C5	1354.8	481.2	4
4	C4	1787.9	204.4	2
	C5	1783.4	187.1	2

## Data Availability

The raw data supporting the conclusions of this article will be made available by the authors on request.
